# Changes in lung cancer-related serum tumor markers in patients with chronic kidney disease and determination of upper reference limit

**DOI:** 10.3389/fonc.2022.1072531

**Published:** 2022-12-08

**Authors:** Qiang Miao, Bei Cai, Qian Niu, Junlong Zhang

**Affiliations:** ^1^ Department of Laboratory Medicine, West China Hospital of Sichuan University, Chengdu, China; ^2^ Research Center of Clinical Laboratory Medicine, West China Hospital of Sichuan University, Chengdu, China

**Keywords:** lung cancer, chronic kidney disease, tumor markers, clinical value, reference range

## Abstract

**Aims:**

To investigate the changes in lung cancer-related serum tumor markers in patients with chronic kidney disease (CKD) and determine the upper reference limit for patients with different stages.

**Methods:**

Included inpatients diagnosed with CKD who did not receive dialysis temporarily in our hospital from March to September 2020. Changes in serum CA125, HE4, CYFRA21-1, SCCA, NSE and ProGRP in CKD patients were analyzed. The non-parametric method was used to estimate the upper reference limit of the above indicators in patients with CKD stages 2-5.

**Results:**

The serum levels of HE4, CYFRA21-1, SCCA, and ProGRP in the CKD group were significantly higher than those in the healthy control group; CA125 and NSE levels were not statistically different. The false positives of SCC, CYFRA21-1, ProGRP, and HE4 increased significantly with the CKD stage. Still, NSE and CA125 did not show a significant increasing trend. Both HE4 and ProGRP have independent upper reference limits from CKD2 to CKD5 stage, namely 220.8 pmol/l and 101.4 pg/ml in the CKD2 stage, 496.7 pmol/l and 168.63 pg/ml in CKD3 stage, 4592.4 pmol/l and 272.8 pmol/l for CKD4 stage, CKD5 stage was 4778.2 pmol/l and 491.6 pmol/l.

**Conclusion:**

This study preliminarily determined the upper reference limits of Lung cancer-related tumor markers in patients with different CKD stages and provided laboratory support for the rational use and interpretation of Lung cancer-related tumor markers in special populations.

## Introduction

Lung cancer is the most common malignant tumor with the highest mortality worldwide, with an estimated 2.20 million new cases and 1.79 million deaths annually (1, 2). It is divided into non-small cell lung cancer (NSCLC) and small cell lung cancer (SCLC) according to different tissue types. Because the early clinical manifestations of lung cancer are not obvious, most patients are already in the advanced stage when diagnosed, and the 5-year survival rate is low. Therefore, improving the early diagnosis rate of lung cancer is the key to improving the 5-year survival rate. A tumor marker is an active substance synthesized and secreted by tumor cells during tumor formation. Detecting serum tumor markers for lung cancer is a non-invasive procedure with important value in diagnosing, monitoring, and evaluating the prognosis of lung cancer.

Lung cancer-related serum markers commonly include carcinoembryonic antigen (CEA), neuron-specific enolase (NSE), cytokeratin fragment 19 (CYFRA21-1), Pro-gastrin-releasing peptide (ProGRP), squamous cell carcinoma antigen (SCCA). Studies have reported that carbohydrate antigen 125 (CA125) is important in the diagnosis and metastasis prediction of lung cancer (3, 4). Recently, studies have also explored human epididymis protein 4 (HE4) as a lung cancer biomarker. The results show that serum HE4 levels are elevated in lung cancer patients of various tissue types, which has an auxiliary role in lung cancer screening (5, 6). The value of Lung cancer-related serum tumor markers in the general population has been widely recognized. However, the concentrations of certain tumor markers are elevated even in the absence of malignancy in chronic kidney disease (CKD) (7). It is mainly due to impaired renal metabolism and excretion, which greatly limits the application of some tumor markers in early diagnosis and treatment monitoring. The utility of tumor markers in diagnosing cancer in patients with renal insufficiency remains controversial (8). In recent years, more and more studies have found that reduced kidney function is associated with a higher risk of cancer. The risk for kidney and lung cancers was higher among those with advanced CKD (9–11). For CKD, patients should be integrated into risk stratification of cancer screening and management. Cancer and CKD both affect many people (1, 12). Issues related to tumor markers in patients with CKD are a matter of concern. It is very important to determine their levels in CKD patients to avoid misinterpretation of Lung cancer-related serum tumor markers in arriving at the diagnosis of lung malignant tumor. Based on the above background, this study intends to explore the level changes and clinical diagnostic value of lung cancer-related serum tumor markers in patients with chronic kidney disease, and to preliminarily determine the upper reference limit for patients with different stages, so as not to confuse with early or preneoplastic stages of malignancy. Provide laboratory support for the rational use of Lung cancer-related serum tumor markers in special populations.

## Materials and methods

### Study population

Selectively included inpatients diagnosed with CKD who did not receive dialysis temporarily in the Department of Nephrology of our hospital from March to September 2020 according to the kidney disease outcomes quality initiative (K/DOQI) guidelines. Patients with related tumors, liver cirrhosis, skin diseases, severe cardiovascular and cerebrovascular diseases, severe infections, pleural and ascites fluids, benign gynecological disorders, and pregnant and lactating women were excluded from the study. A total of 729 eligible CKD patients (415 males and 314 females) with an average age of 49(38, 60) years were enrolled, including 172 CKD2 stage patients, 169 CKD3 stage patients, 167 CKD4 stage patients, and 221 CKD5 stage patients. At the same time, 94 healthy subjects (47 males and 47 females) were included as controls, with an average age of 42 (36, 48). This study protocol was approved by the Ethics Committee of the West China Hospital, Sichuan University (No. 2020-823). All methods were performed following the relevant guidelines and regulations. All participants obtained informed consent.

### Sample collection and measurements

3-5 mL of fasting venous blood was collected from the research subjects, centrifuged at 1 200 × g for 10 min to obtain serum, and stored at -80°C until measurement. Serum CA125, HE4, NSE, CYFRA21-1, ProGRP and SCCA levels were detected by Roche Cobas e801 electrochemiluminescence immunoassay analyzer, and serum urea, creatinine and cystatin C levels were detected by Roche Cobas c701 automatic biochemical analyzer. All assays were performed using Roche Diagnostics (Mannheim, Germany) kits and processed according to the manufacturer’s instructions. The testing items involved in this study participated in the quality evaluation activities organized by the clinical laboratory center of the National Health Commission and the College of American Pathologists every year. The results were satisfactory.

The estimated glomerular filtration rate (eGFR) was calculated using the Chronic Kidney Disease Epidemiology Collaboration(CKD-EPI) equation (12). According to eGFR≥90 ml·min^-1^·1.73 m^-2^, eGFR 60~<90 ml·min^-1^·1.73 m^-2^, eGFR30~<60 ml·min^-1^·1.73 m^-2^, eGFR15~<30 ml·min^-1^·1.73 m^-2^ and eGFR<15 ml·min^-1^·1.73 m^-2^ divided CKD patients into CKD1, CKD2, CKD3, CKD4 and CKD5 stages. According to the laboratory’s current reference intervals, patient results in this study who were outside the reference intervals but were excluded tumor by clinicians based on imaging and other relevant tests were assumed to be false positives. The reference intervals of our laboratory were CYFRE21-1<3ng/ml, SCC<2.7ng/ml, NSE<20.4ng/ml, ProGRP<65.7pg/ml, CA125(females) 0~49 years<47 U/ml, 50 years and above<25 U/ml, CA125(males)<24 U/ml; HE4(female) 18~39 years<60.5 pmol/L, 40~49 years<76.2 pmol/L, 50~59 years<74.3 pmol/L, 60~69 years<82.9 pmol/L, 70 years and above<104 pmol/L.

### Upper reference limit calculation

The reference upper limit of patients with different CKD stages was set using the non-parametric method to take the 97.5th quantile as the upper limit. When the concentration comparison between other CKD groups is significantly different, and the results of the normal deviation test (z-test) indicate that the reference range needs to be set according to the subgroup, the corresponding upper reference limit of the subset is set. The formula for the z-test is as follows (13):


$$Z=\frac{\left|\bar{X}1-\bar{X}2\right|}{{\left[\left(\frac{S_{1}^{2}}{N_{1}}\right)+\left(\frac{S_{2}^{2}}{N_{2}}\right)\right]}^{\frac{1}{2}}},\;Z^{*}=3{\left[(N1+N2)/240\right]}^{\frac{1}{2}},\;(N1\geq 120,\;N2\geq 120)$$

X̅1 and X̅2 are the practical means of the two subgroups, S1 and S2 are the observed variances, and N1 and N2 are the number of reference values in each subclass. If the calculated Z exceeds Z*, they recommend partitioning.

### Statistical analysis

All statistical analyses were performed with SPSS 23.0 software (SPSS Inc., Chicago, IL, USA). Values are presented as the mean ± standard deviation for data normally distributed or median and interquartile range for data that were non-normally distributed for continuous variables and number (%) for categorical variables. Use the analysis of covariance (ANCOVA) to control the effects of age-confounding variables. Means of two continuous normally distributed variables were compared by independent samples Student’s t-test. Mann-Whitney U and Kruskal-Wallis tests were used to compare the means of two and multi-group variables that are not normally distributed. A Dunn-Bonferroni test was used for *post hoc* comparisons. The Pearson test was used for correlation analysis. When appropriate, the frequencies of categorical variables were compared using the Pearson chi-square test or Fisher s exact test. For all comparisons, P<0.05 was considered statistically significant.

## Results

### Clinical characteristics of study subjects

The general clinical and biochemical characteristics of the study subjects are summarized in **Table 1**. The major clinical etiological categories of renal disease in CKD patients included glomerulonephritis, diabetic nephropathy, and hypertensive nephropathy. Patients with CKD were significantly older than healthy controls. Still, there was no significant difference in gender between the two groups. In addition, renal function-related indicators of urea, creatinine, and cystatin C in CKD patients were significantly increased, while eGFR was decreased considerably. We also analyzed the levels of tumor markers between CKD patients caused by autoimmune factors and CKD patients caused by other factors, as well as healthy controls. The results are shown in **Table S1** (supplementary materials). According to the data, there is no significant difference between CKD caused by autoimmune factors and CKD caused by other factors. Before and after the removal of autoimmune factors in CKD patients, there was almost no significant change in the study subjects’ median level of tumor markers (**Tables 2**, **S1**). It suggests that renal dysfunction may play a major role in influencing the level of tumor markers compared with other underlying diseases, such as autoimmune diseases.

**Table 1:** Analysis of general clinical characteristics of the healthy control group and CKD group.

Parameters	CKD group (n=729)	control group(n=94)	*P*
Age(year)	49 (38, 60)	42 (36, 48)	0.000
Gender (male/female)	415/314	47/47	0.203
Clinical kidney disorders			
Hypertensive nephropathy	134 (18.3%)	—	—
Glomerulonephritis	235 (32.2%)	—	—
Diabetic nephropathy	205 (28.1%)	—	—
Autoimmune kidney disease	70 (9.6%)	—	—
Others	85 (11.6%)	—	—
Ur (mmol/L)	11.1(6.7, 18.5)	4.4(3.8, 5.2)	0.000
Cr (umol/L)	192(117, 436.5)	67.5(58.8, 79.3)	0.000
Cysc (mg/L)	2.26(1.42, 4.07)	0.76(0.71, 0.84)	0.000
eGFR (ml·min^-1^·1.73 m^-2^)	28.2(11.8, 58.4)	105.5(102.8, 108.5)	0.000

Data are summarized as median (interquartile range) for continuous variables or as a number with proportion for categorical variables. CKD: chronic kidney disease; Ur: urea; Cr: creatinine; Cysc: cystatin C; eGFR: estimated glomerular ﬁltration rate.

**Table 2:** Serum tumor marker levels in the control group and CKD group.

Tumor makers	CKD group(n=729)	Control group(n=94)	*P*	Adjust P*
CA125 (U/mL)	15.5 (9.9, 26.9)	12.2 (9.6, 16.3)	0.000	0.224
HE4 (pmol/L)	286 (120.5, 959)	44.7 (39.5, 51.2)	0.000	0.000
CYFRA21-1 (ng/ml)	3.75 (2.63, 5.37)	1.46 (1.17, 1.82)	0.000	0.000
SCCA (ng/ml)	1.94 (1.23, 3.4)	0.98 (0.7, 1.38)	0.000	0.000
NSE (ng/ml)	14.3 (11.5, 18.2)	15.5 (13.7, 17.3)	0.016	0.380
ProGRP(pg/ml)	96.1 (60.9, 164.5)	38.1 (33.0, 47.4)	0.000	0.000

Data are summarized as median (interquartile range) for continuous variables. CKD: chronic kidney disease; CA125: carbohydrate antigen 125; HE4: human epididymis protein 4; CYFRA21-1: cytokeratin fragment 19; SCCA: squamous cell carcinoma antigen; NSE: neuron-specific enolase; ProGRP: pro-gastrin-releasing peptide.

* Use the analysis of covariance (ANCOVA) to control the effects of age confounding variables.

### Lung cancer-related serum tumor marker levels

The serum Lung cancer-related tumor marker levels in CKD patients and healthy controls are shown in **Table 2**. The serum levels of HE4, CYFRA21-1, SCCA, and ProGRP in the CKD group were significantly higher than those in the healthy control group before and after controlling the effects of age confounding variables. However, serum CA125 and NSE levels were not statistically different between the two groups after adjustment for age confounding factors. We further compared the levels of Lung cancer-related serum tumor markers between the healthy control group and other CKD-stage groups. The results are shown in **Table 3**. Serum CA125 and CYFRA21-1 levels in patients with advanced CKD (i.e., CKD stages 4 and 5) were significantly higher than in healthy controls, and patients with CKD stages 2 and 3. In addition, the serum CYFRA21-1 increased dramatically in CKD stages 2 and 3 compared with the healthy control group. Serum SCCA levels were significantly different during CKD stages 3, 4 and 5. They were significantly higher than healthy controls and CKD stage 2 patients. Compared with healthy controls, the serum levels of HE4 and ProGRP in CKD patients increased considerably, showing a gradually increasing trend from CKD stage 2. The concentration levels were statistically different in different CKD stages. In contrast, healthy controls and patients with varying stages of CKD had comparable NSE levels.

**Table 3:** Analysis of serum tumor marker levels in patients with different stages of CKD.

Tumor makers	control group(n=94)	CKD2(n=172)	CKD3(n=169)	CKD4(n=167)	CKD5(n=221)	*P*
CA125 (U/mL)	12.2 (9.6, 16.3)	13.4 (8.7, 19.9)	14.3 (9.5, 22.1)	19.3 (12, 64)^abc^	17.1 (10.2, 34)^abc^	0.000
CYFRA21-1 (ng/ml)	1.46 (1.17, 1.81)	2.48 (1.96, 3.26)^a^	3.29 (2.51, 4.13)^ab^	4.23 (3.33, 6.01)^abc^	5.05 (3.92, 6.62)^abc^	0.000
SCCA (ng/ml)	0.98 (0.7, 1.38)	1.22 (0.86, 1.76)	1.65 (1.11, 2.45)^ab^	2.17 (1.46, 4.02)^abc^	3.31 (1.94, 5.39)^abcd^	0.000
NSE (ng/ml)	15.5 (13.7, 17.3)	14 (11.8, 17.4)	13.3 (10.7, 16.9)	14.3 (11.4, 18)	15.4 (11.9, 20.5)	0.000
HE4 (pmol/L)	44.6 (39.5, 51.2)	89.1 (74.5, 110)^a^	151 (124, 234)^ab^	394 (244, 721)^abc^	1332 (823.5, 2156.5)^abcd^	0.000
ProGRP(pg/ml)	38.1 (33, 47.4)	52.2 (45.1, 65.3)^a^	76.5 (60.7, 98.1)^ab^	108 (83.1, 144)^abc^	203 (147, 268.5)^abcd^	0.000

Data are summarized as median (interquartile range) for continuous variables. CKD: chronic kidney disease; CA125: carbohydrate antigen 125; HE4: human epididymis protein 4; CYFRA21-1: cytokeratin fragment 19; SCCA: squamous cell carcinoma antigen; NSE: neuron-specific enolase; ProGRP: pro-gastrin-releasing peptide. ^a^ P<0.05 versus control group; ^b^ P<0.05 versus CKD2; ^c^ P<0.05 versus CKD3; ^d^ P<0.05 versus CKD4.

### False positive rates

The false positive analysis of Lung cancer-related tumor markers in patients with different CKD stages and healthy controls is summarized in **Table 4**. The false positives of blood SCC, CYFRA21-1, ProGRP and HE4 increased significantly with the CKD stage. Still, NSE and CA125 did not show a significant increasing trend. Among them, the false positive rates of CYFRA21-1, ProGRP, and HE4 in patients with CKD stage 3-5 were all above 60%, which was significantly higher than that of healthy controls and CKD stage 2.

**Table 4:** The false positive* rate analysis of tumor markers in patients with different stages of CKD.

Tumor makers	control group(n=94, female 47)	CKD2(n=172, female 74)	CKD3(n=169, female 73)	CKD4(n=167, female 74)	CKD5(n=221, female 93)	*P*
CYFRA21-1, n(%)	5_a_ (5.3)	58_b_ (33.7)	103_c_ (60.9)	140_d_ (83.8)	195_d_ (88.2)	0.000
SCCA, n(%)	4_a_ (4.3)	17_a,b_ (9.9)	32_b_ (18.9)	69_c_ (41.3)	131_d_ (59.3)	0.000
NSE, n(%)	11_a,b_ (11.7)	25_a,b_ (14.5)	15_b_ (8.9)	30_a,b_ (18)	56_a_ (25.3)	0.000
ProGRP, n(%)	0_a_ (0)	40_b_ (23.3)	113_c_ (66.9)	147_d_ (88)	218_e_ (98.6)	0.000
CA125, n(%)	4_a_ (4.3)	17_a_ (9.9)	17_a_ (10.1)	62_b_ (37.1)	65_b_ (29.4)	0.000
HE4^#^, n(%)	1_a_ (2.1)	49_b_ (66.2)	70_c_ (95.9)	74_c_ (100)	93_c_ (100)	0.000

#Only analyze the false positive rate among females. CKD: chronic kidney disease; CA125: carbohydrate antigen 125; HE4: human epididymis protein 4; CYFRA21-1: cytokeratin fragment 19; SCCA: squamous cell carcinoma antigen; NSE: neuron-specific enolase; ProGRP: pro-gastrin-releasing peptide.

*Positive is defined as greater than the reference range.The reference range of the laboratory: CYFRE21-1<3ng/ml, SCC<2.7ng/ml, NSE<20.4ng/ml, ProGRP<65.7pg/ml, CA125(female): (0~49 years)<47U/ml, 50 years and above <25U/ml, CA125(male) <24U/ml; HE4(female):(18~39 years)<60.5pmol/L, (40~49 years)<76.2pmol/L, (50~59 years)<74.3pmol/L, (60~69 years)<82.9pmol/L, (70 years and above)<104pmol/L.

a, b, c, d, e: Each subscript letter indicates a subset of the grouping category. At the 0.05 level, the column ratios of these categories are not significantly different from each other.

### Correlation analysis

**Figure 1:**
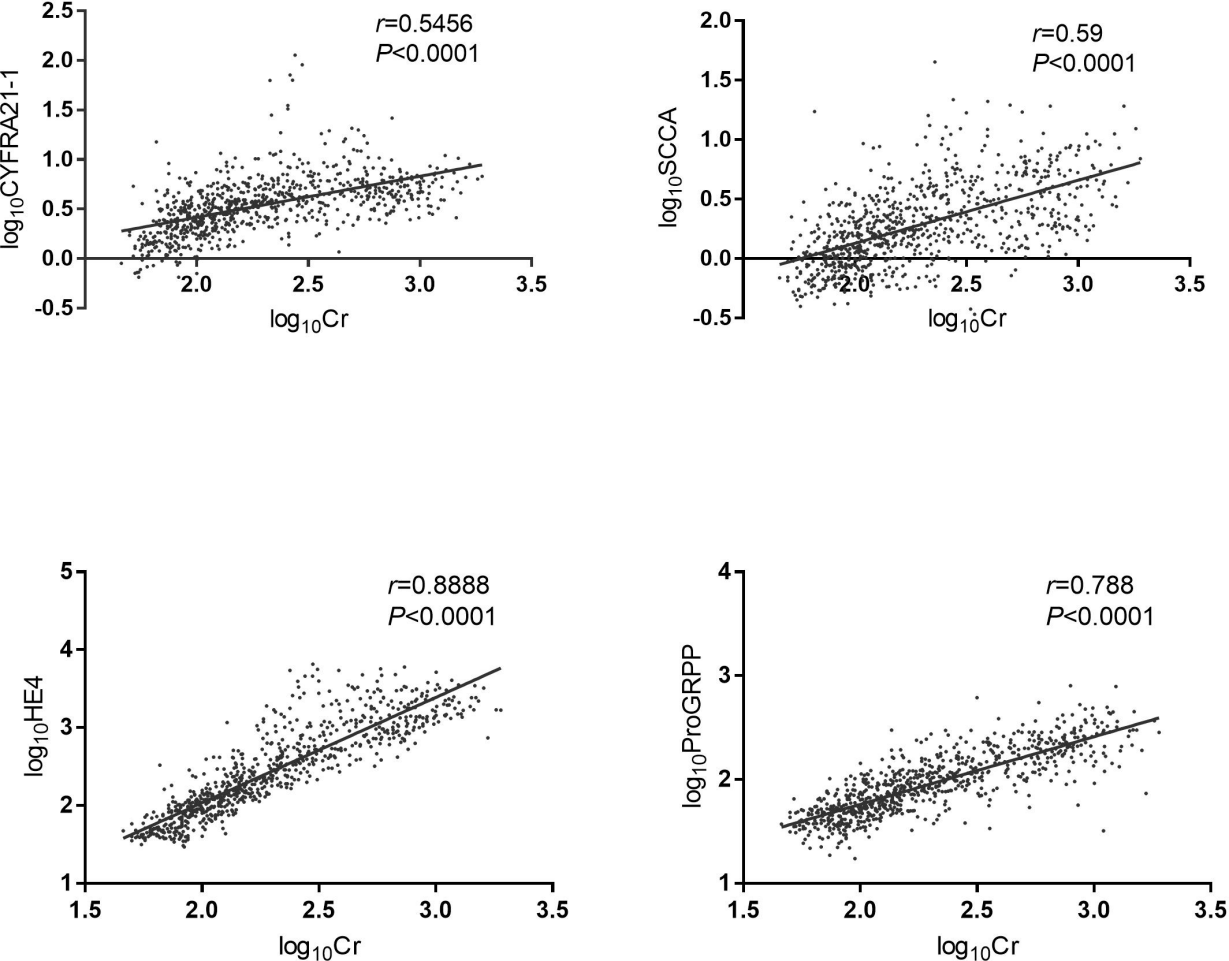
Correlation analysis of lung cancer-related tumor markers and serum creatinine. The data were analyzed by Person correlation after common logarithmic transformation. HE4: human epididymis protein 4; CYFRA21-1: cytokeratin fragment 19; SCCA: squamous cell carcinoma antigen; ProGRP: pro-gastrin-releasing peptide; Cr: creatinine. Log_10_HE4: the logarithm of HE4 concentration to the base 10. Log_10_CYFRA21-1: the logarithm of CYFRA21-1 concentration to the base 10. Log_10_SCCA: the logarithm of SCCA concentration to the base 10. Log_10_ProGRP: the logarithm of ProGRP concentration to the base 10. Log_10_Cr: the logarithm of creatinine concentration to the base 10.

The results of the correlation analysis are shown in **Figure 1**. The common logarithm of the levels of lung cancer-related serum tumor markers HE4, SCC, ProGRP and CYFRA21-1 in CKD patients was significantly positively correlated with creatinine.

### The upper reference limit in different CKD stages

According to the results of the pairwise comparison between the groups in **Table 3** and the Z-test formula, the upper reference limits for different stages are summarized in **Table 5**. The upper reference limit of CA125 in the CKD4-5 stage was 358.1 U/ml, and the upper reference limit of CYFRA21-1 in CKD2, CKD3 and CKD4-5 stage were 6.99 ng/ml, 9.19 ng/ml and 22.24 ng/ml, respectively. The upper reference limits of SCCA in CKD3 and CKD4-5 were 5.17ng/ml and 13.97ng/ml, respectively. Both HE4 and PaoGRP have independent upper reference limits from CKD2 to CKD5 stage, namely 220.8 pmol/l and 101.4 pg/ml in the CKD2 stage, 496.7 pmol/l and 168.63 pg/ml in CKD3 stage, 4592.4 pmol/l and 272.8 pmol/l for CKD4 stage, CKD5 stage was 4778.2 pmol/l and 491.6 pmol/l.

**Table 5:** The reference upper limit defined by non-parametric method (P_97.5_) in patients with different stages of CKD.

Tumor makers	CKD2(n=172)	CKD3(n=169)	CKD4(n=167)	CKD5(n=221)
CA125 (90% CI, U/ml)	–	–	358.1 (259.65, 505.43)	
CYFRA21-1 (90% CI, ng/ml)	6.99 (5.46, 9.15)	9.19 (7.53, 11.1)	22.24 (16.2, 42.78)	
SCCA (90% CI, ng/ml)	–	5.17 (4.56, 6.27)	13.97 (11.28, 19.2)	
HE4 (90% CI, pmol/l)	220.8 (162.7, 283)	496.7 (418.9, 1056)	4592.4 (3216.4, 5586.4)	4778.2 (3792, 5443)
ProGRP (90% CI, pg/ml)	101.4 (89.5, 110)	168.63 (148.73, 193)	272.8 (236.8, 298)	491.6 (438.27, 551)

P_97.5_: 97.5th percentile; 90% CI: confidence interval; CKD: chronic kidney disease; CA125: carbohydrate antigen 125; HE4: human epididymis protein 4; CYFRA21-1: cytokeratin fragment 19; SCCA: squamous cell carcinoma antigen; ProGRP: pro-gastrin-releasing peptide.

## Discussion

Impaired kidney function is not only associated with an increased risk of cancer but is also a feature of many cancer patients. Serum tumor markers are protein substances secreted or shed by tumor cells. Their content is extremely low in healthy people. Elevated serum concentrations often indicate the occurrence of malignant tumors. Each tumor marker has a variable profile of usefulness for screening, determining diagnosis and prognosis, assessing response to therapy, and monitoring for cancer recurrence. We are still unclear about the body’s metabolic process of tumor markers. In clinical applications, it has been found that in addition to related tumors, their concentrations will also be abnormal in the menstrual cycle, pregnancy, metabolism, inflammation, liver function and (or) renal function abnormalities (14). CKD is a disease that seriously endangers human health, with a prevalence of 10.8% in the adult population in China (15). Regardless of the occurrence of malignant tumors, the concentrations of some tumor markers in CKD patients will be higher than those in healthy subjects. However, the impaired renal function does not alter all tumor markers (7). Which indicators will be affected and to what extent is unclear. In this study, the serum tumor markers related to lung cancer levels in CKD patients were analyzed. It was found that the levels of HE4, CYFRA21-1, SCCA and ProGRP were significantly increased in CKD patients. In addition, with the increase in the CKD stage, the false positive rate of the four tumor markers gradually increased significantly. The level of CA125 was not affected in the early stage of CKD and only increased significantly in the late stage. In contrast, the level of NSE was hardly affected by renal function.

Renal insufficiency, dialysis, and kidney transplantation were associated with an increased risk of lung cancer. However, studies have shown that commonly used serum Lung cancer-related tumor markers are elevated in patients with decreased renal function. In a study by Jianzhong Chen et al. (16) in patients with diabetic nephropathy, serum SCCA and CYFRA21-1 levels were significantly increased, while NSE was unaffected. It is worth noting that this study only investigated renal insufficiency due to diabetic nephropathy, while we further analyzed patients with CKD due to various causes and obtained the same results. K. Kamata et al. (17) reported elevated serum proGRP levels in patients with renal insufficiency. Linda Hertlein et al. (18) Among other benign diseases, including lung and ovarian cancer, HE4 concentrations were highest in women and men with renal failure. Our study also found that serum HE4 and ProGRP levels were significantly increased in non-tumor CKD patients. With the aggravation of CKD staging, the average levels of each stage increased, and their levels were significantly different in each stage, which adds new and further evidence to the previous studies. The results of studies on serum CA125 concentrations in CKD patients vary. Some studies have shown that serum CA125 levels in CKD patients are higher than in healthy controls. Still, there is no significant correlation with eGFR (19). Some studies have found significant differences in CAl25 levels in patients with different stages of CKD. In contrast, other studies have shown no exact relationship between CKD stages and CAl25 levels (20). This study did not find significant differences in CA125 levels between CKD patients and healthy controls after adjusting for age confounding variables. But further observed that CA125 levels in different CKD stages were not affected in the early stage of CKD, and only significantly increased in the late stage.

In this study, we found that the changes in HE4, ProGRP, SCC, and CYFRA21-1 in CKD patients had a certain relationship with the CKD stage. The correlation analysis also showed that their levels were significantly positively correlated with creatinine. The false-positive rate of HE4 was also significantly better than that of NSE and CA125. The above results showed that the degree of increase of indexes such as HE4 was closely related to the severity of renal damage. Some indexes showed an increasing trend in the early stage of renal damage.

In addition, to more reasonably interpret the results of Lung cancer-related tumor markers in CKD patients, early detection of tumors can prevent missed diagnosis and missed treatment while simultaneously avoiding the waste of medical resources caused by misdiagnosis and mistreatment. Based on some principles established by the reference interval, this study determined the upper reference limit for CKD patients of different stages so that clinicians can make timely and accurate judgments on the tumor marker results of CKD patients. Unfortunately, due to the insidious nature of the onset of CKD, patients are often not at an early stage when they seek medical attention. Therefore, no CKD stage 1 patients were included in this study. The upper reference limit of the above indicators in patients with CKD1 stage cannot be determined. It is impossible to assess whether indicators such as HE4 are more advantageous in diagnosing earlier renal injury.

Moreover, this study estimates the reference range of CKD patients based on the included research subjects. The results have certain limitations due to the influence of the sample size. Further studies with large samples are needed to determine a more accurate reference range.

## Conclusion

Serum levels of HE4, ProGRP, CYFRA21-1, SCC and CA125 were closely related to renal function, while NSE levels were not. This study preliminarily determined the upper reference limits of Lung cancer-related tumor markers in patients with different CKD stages and provided laboratory support for the rational use and interpretation of Lung cancer-related tumor markers in special populations.

## Data availability statement

The original contributions presented in the study are included in the article/**Supplementary Material**. Further inquiries can be directed to the corresponding author.

## Ethics statement

The studies involving human participants were reviewed and approved by the Ethics Committee of the West China Hospital, Sichuan University. The patients/participants provided their written informed consent to participate in this study.

## Author contributions

All authors contributed to the study’s conception and design. QM, BC, and QN performed material preparation, data collection and analysis. JZ and QM wrote the first draft of the manuscript. All authors commented on previous versions of the manuscript. All authors contributed to the article and approved the submitted version.
