# Supplementary material for: Changes in lung cancer-related serum tumor markers in patients with chronic kidney disease and determination of upper reference limit
Source: Front Oncol. 2022 Dec 8;12:1072531. doi: 10.3389/fonc.2022.1072531 (PMC9772264; doi:10.3389/fonc.2022.1072531)
Supplement: Supplementary file 1 [file Table_1.docx]

**Changes in Lung cancer-related serum tumor markers in patients with chronic kidney disease and determination of upper reference limit**

Qiang Miao, Bei Cai, Qian Niu, Junlong Zhang#

| **Table S1 Analysis of serum tumor markers in patients with CKD caused by different etiologies** | | | | | | |
| --- | --- | --- | --- | --- | --- | --- |
| Tumor makers | Other factors group (n=659) | Autoimmune factor group (n=70) | Control group(n=94) | *P1* | *P2* | *P3* |
| CA125 (U/mL) | 15.4 (9.8, 27.4) | 16.3 (11.3, 24.1) | 12.2 (9.6, 16.3) | 0.017 | 0.001 | 1.000 |
| HE4 (pmol/L) | 302 (124, 1041) | 152.5 (102, 442.5) | 44.7(39.5, 51.2) | 0.000 | 0.000 | 0.015 |
| CYFRA21-1 (ng/ml) | 3.72 (2.6, 5.37) | 3.82 (2.98, 5.32) | 1.46(1.17, 1.82) | 0.000 | 0.000 | 1.000 |
| SCCA (ng/ml) | 1.97 (1.26, 3.66) | 1.78 (1.09, 3.04) | 0.98(0.7, 1.38) | 0.000 | 0.000 | 0.459 |
| NSE (ng/ml) | 14.3 (11.5, 18.3) | 14.1 (11.8, 17.1) | 15.5(13.7, 17.3) | 0.146 | 0.056 | 1.000 |
| ProGRP(pg/ml) | 96.8 (62.1, 167) | 79.5 (53.8, 137) | 38.1(33.0, 47.4) | 0.000 | 0.000 | 0.268 |
| Data are summarized as median (interquartile range) for continuous variables. CKD: chronic kidney disease; CA125: carbohydrate antigen 125; HE4: human epididymis protein 4; CYFRA21-1: cytokeratin fragment 19; SCCA: squamous cell carcinoma antigen; NSE: neuron-specific enolase; ProGRP: pro-gastrin-releasing peptide.  *P1,* Comparison of autoimmune disease factor group with healthy control group. *P2,* Comparison of other factor groups with healthy controls*. P3,* Comparison of other factor groups and autoimmune factor groups. | | | | | | |
